# Supplementary material for: Breast and pancreatic cancer interrupt IRF8-dependent dendritic cell development to overcome immune surveillance
Source: Nat Commun. 2018 Mar 28;9:1250. doi: 10.1038/s41467-018-03600-6 (PMC5871846; doi:10.1038/s41467-018-03600-6)
Supplement: Supplementary file 1 — Supplementary Information(PDF 3958 kb) [file 41467_2018_3600_MOESM1_ESM.pdf]

SUPPLEMENTARY INFORMATION

Breast and Pancreatic Cancer Interrupt IRF8-Dependent Dendritic Cell Development to Overcome Immune Surveillance

Meyer et al.

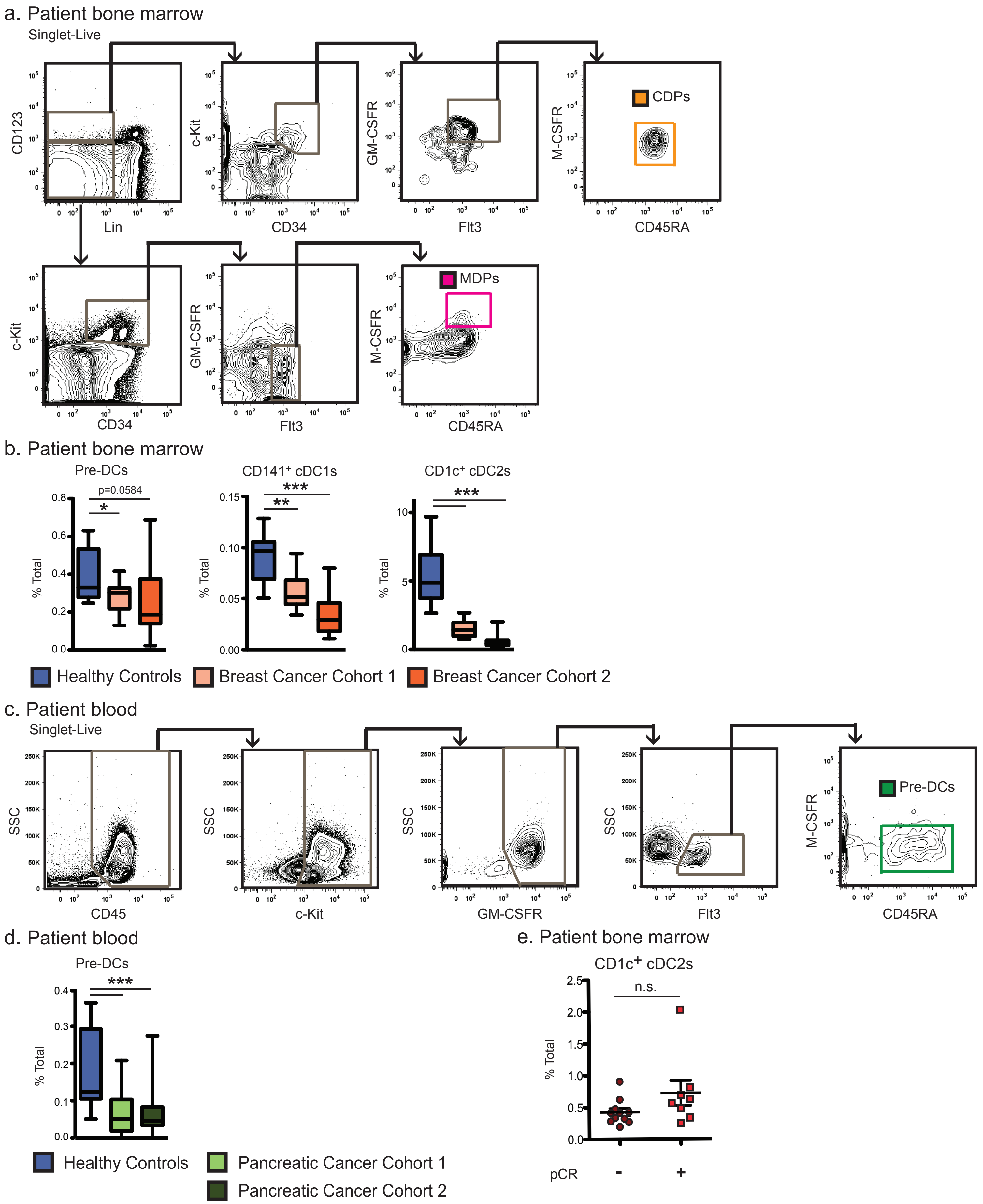

Supplementary Figure 1. (**a**) Representative gating strategy for human BM MDPs and CDPs. (**b**) Frequency of BM pre-DCs, CD141^{+} cDC1s, and CD1c^{+} cDC2s in baseline breast cancer patients relative to healthy controls. Data are from breast cancer cohorts 1 and 2; healthy controls, n=12; breast cancer cohort 1, n=10; breast cancer cohort 2, n=21. (**c**) Representative gating strategy for human blood pre-DCs.(**d**) Frequency of blood pre-DCs in baseline pancreatic cancer patients relative to healthy controls. Data are from pancreatic cancer cohorts 1 and 2. (**e**) Frequency of BM CD1c^{+} cDC2s in breast cancer patients achieving pathological complete response (pCR) relative to patients who did not achieve pathological complete response in breast cancer cohort 2; n=18. Box plot; error bars represent mean +/- s.e.m.; *p<0.05, **p<0.01, ***p<0.001, n.s. not significant by unpaired two-sided Student's t test.

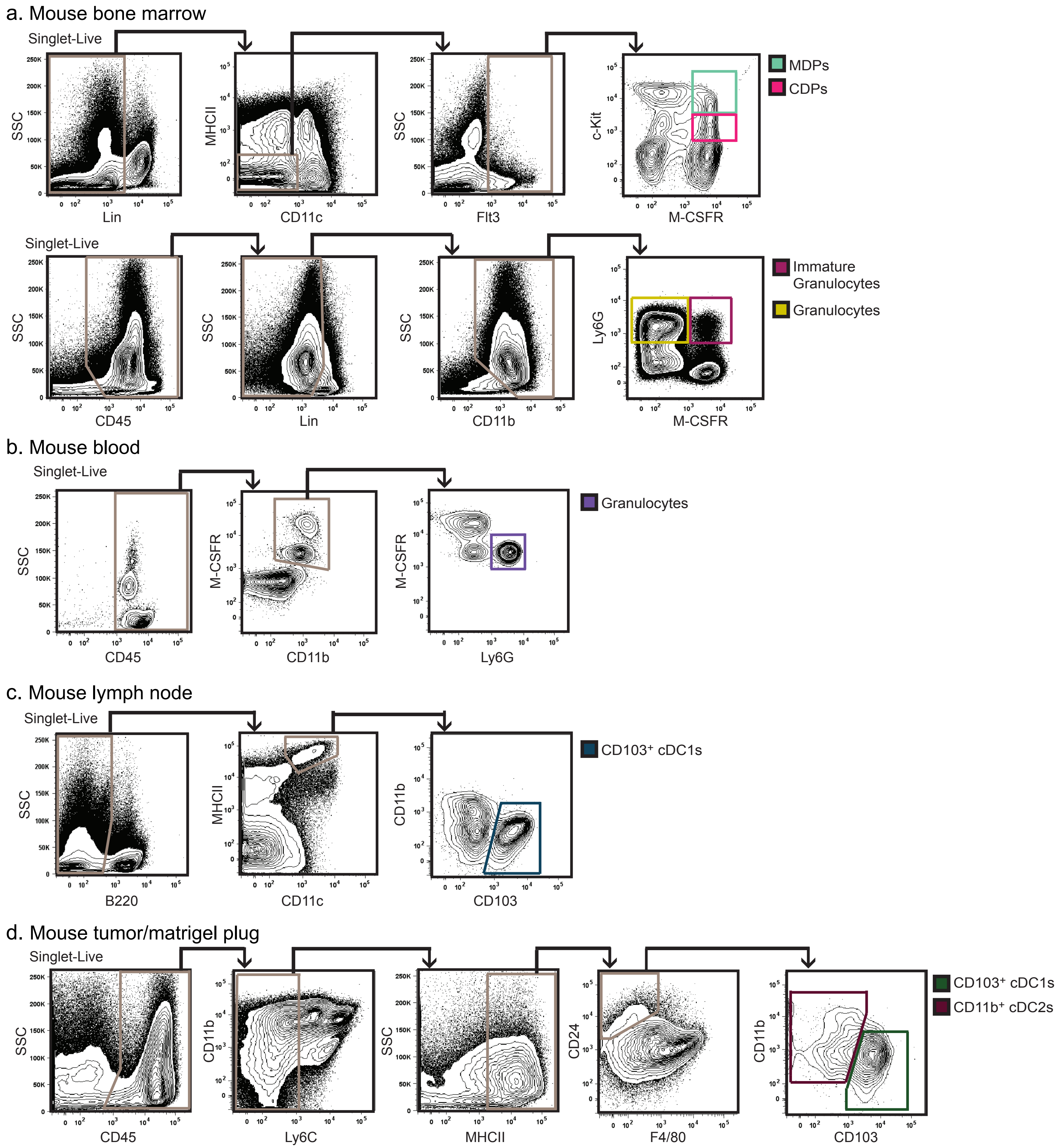

Supplementary Figure 2. Representative gating strategy for (**a**) mouse BM MDPs, CDPs, immature granulocytes, and granulocytes; (**b**) mouse blood granulocytes; (**c**) mouse lymph node CD103+ cDC1s; (**d**) mouse tumor and matrigel plug CD103+ cDC1s and CD11b+ cDC2s.

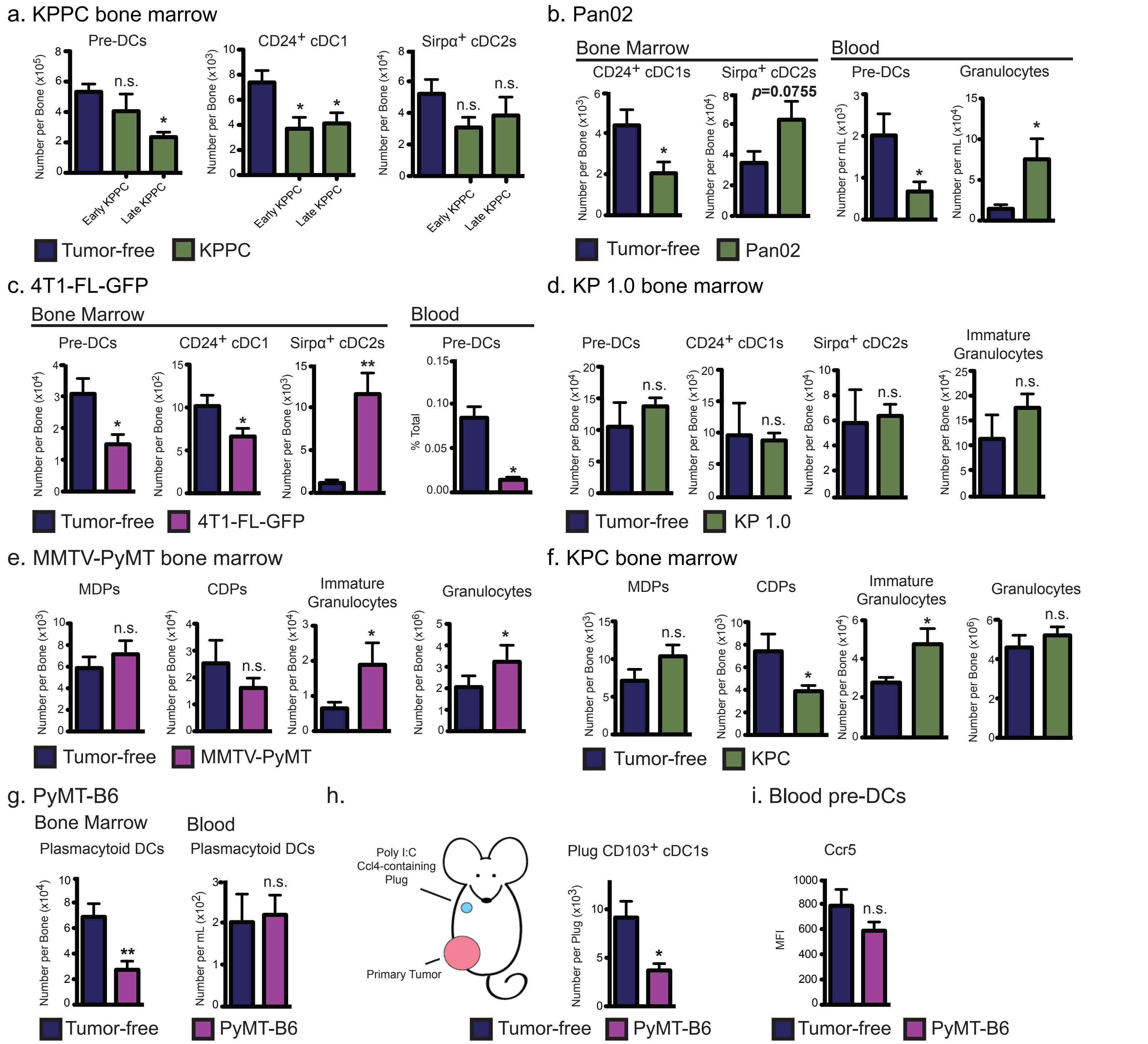

Supplementary Figure 3. (**a**) Number of BM pre-DCs, CD24⁺ cDC1s, and Sirpα⁺ cDC2s in early (1-month-old) and late (end-stage, 1.5-month-old) accelerated genetic mouse model of pancreatic cancer (KPPC) relative to tumor-free controls; n=5/group. (**b**) Number of BM CD24⁺ cDC1s and Sirpα⁺ cDC2s and blood pre-DCs and granulocytes in mice with end-stage orthotopic Pan02 pancreatic tumors relative to tumor-free controls; n=6/group. Data are representative of two independent experiments. (**c**) Number of BM pre-DCs, CD24⁺ cDC1s, and Sirpα⁺ cDC2s, and blood frequency of pre-DCs in mice with end-stage orthotopic 4T1-FL-GFP mammary tumors relative to tumor-free controls; n=6/group. (**d**) Number of BM pre-DCs, CD24⁺ cDC1s, and Sirpα⁺ cDC2s in mice with end-stage orthotopic KP 1.0 pancreatic tumors relative to tumor-free controls; n=6/group. Number of BM MDPs, CDPs, immature granulocytes, and granulocytes from end-stage genetic mouse models of (**e**) breast cancer (MMTV-PyMT) and (**f**) pancreatic cancer (KPC) relative tumor-free controls; tumor-free MMTV-PyMT controls, n=6; MMTV-PyMT, n=6; tumor-free KPC controls, n=7; KPC, n=8. (**g**) Number of BM and blood plasmacytoid DCs in mice with end-stage orthotopic PyMT-B6 tumors. (**h**) Mice with 1.0-cm diameter orthotopic PyMT-B6 tumors and tumor-free controls were implanted with matrigel plugs containing poly I:C and Ccl4 in the upper mammary fat pad. Number of CD103⁺ cDC1s in the plug 10 days after implant; n=6/group. (**i**) Ccr5 expression in blood pre-DCs in mice with end-stage orthotopic PyMT-B6 tumors. End-stage for each model is defined in the methods. Error bars represent mean +/- s.e.m.; *p<0.05, **p<0.01, ***p<0.001, n.s. not significant by unpaired two-sided Student's t test.

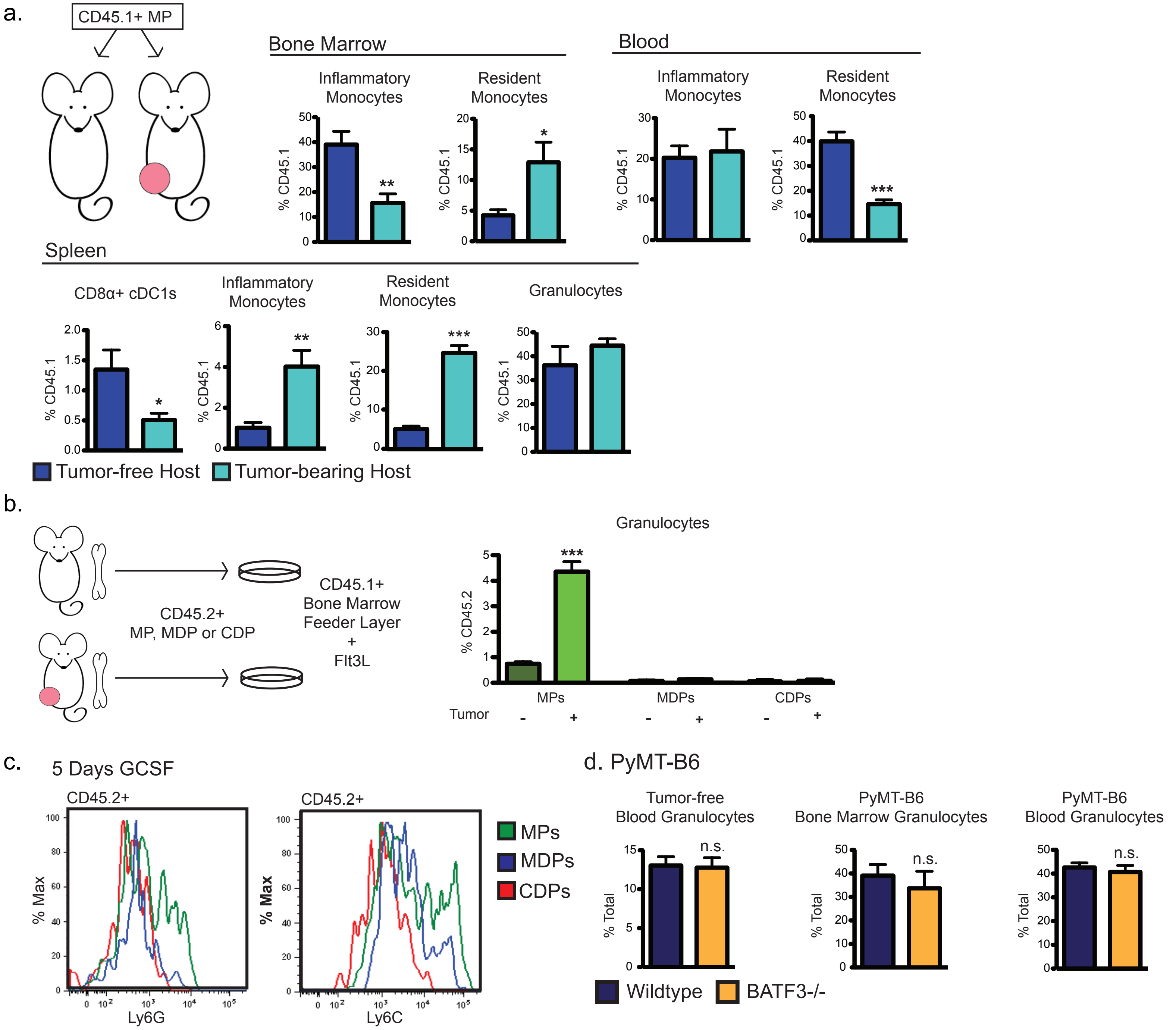

Supplementary Figure 4. (**a**) CD45.1^{+}Lin^{-}Sca1^{-}cKit^{+} MPs were transferred into orthotopic PyMT-B6 mammary tumor-bearing mice or tumor-free controls. Frequency of BM inflammatory monocytes and resident monocytes; blood inflammatory monocytes and resident monocytes; and spleen CD8α^{+} cDC1, inflammatory monocytes, resident monocytes and granulocytes, as frequency of CD45.1; n=5/group. (**b**) CD45.2^{+}Lin^{-}Sca1^{-}cKit^{+} MPs, CD45.2^{+} MDPs, and CD45.2^{+} CDPs were isolated from end-stage orthotopic PyMT-B6 tumor-bearing or tumor-free donors. Progenitors were cultured on CD45.1^{+} BM feeder culture for 5 days in the presence of 100 ng/ml Flt3L. Final cultures were analyzed for granulocytes (Live CD45.2^{+}CD45.1^{-}CD11b^{+}Ly6G^{+}). Data are representative of three independent experiments consisting of three wells per condition. (**c**) Representative plots of Ly6G and Ly6C expression in sorted MPs, MDPs and CDPs after 5 days of culture with GCSF. (**d**) Percentage of granulocytes in the blood of tumor-free wild-type and BATF3$^{-/-}$. Percentage of granulocytes in the BM and blood of wild-type and BATF3$^{-/-}$ mice with end-stage PyMT-B6 orthotopic mammary tumors; n=6/group. End-stage is defined in the methods. Error bars represent mean +/- s.e.m.; *p<0.05, **p<0.01, ***p<0.001, n.s. not significant by unpaired two-sided Student's t test.

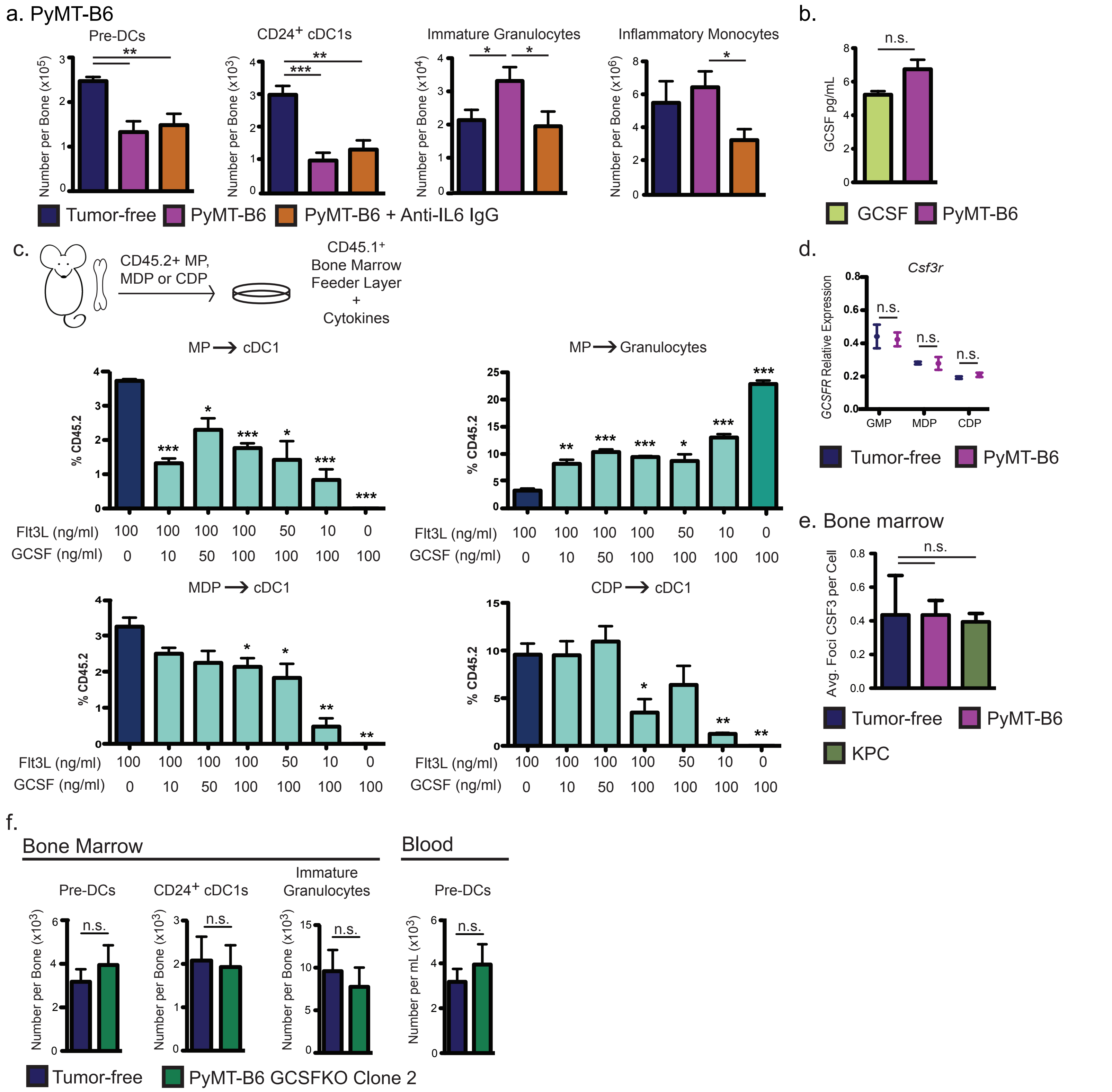

Supplementary Figure 5. (**a**) Tumor-free, orthotopic PyMT-B6 tumor-bearing end-stage mice and orthotopic PyMT-B6 tumor-bearing end-stage mice treated for 2 weeks with 500 µg anti-IL6 IgGs 3x/week were analyzed. Numbers of BM pre-DCs, CD24^{+} cDC1s, immature granulocytes, and inflammatory monocytes (CD45^{+}CD11b^{+}CD115^{+}Ly6C^{+}Ly6G^{-}); n=6/groups. (**b**) GCSF serum concentration in mice treated with 2 µg GCSF for 5 days and mice bearing end-stage PyMT-B6 tumors. (**c**) CD45.2^{+} Lin^{-}Sca1^{-}cKit^{+} MPs, MDPs and CDPs were cultured on CD45.1^{+} BM feeder culture in the presence of indicated GCSF and Flt3L concentration for 5 days. Final cultures were analyzed for cDC1s (Live CD45.2^{+}CD45.1^{-}MHCII^{+}CD11c^{+}Sirpα^{-}CD24^{+}) and granulocytes (Live CD45.2^{+}CD45.1^{-}CD11b^{+}Ly6G^{+}). Data are representative of three independent experiments consisting of three wells per condition. (**d**) RT-qPCR analysis of BM GMPs, MDPs, and CDPs sorted from mice bearing end-stage orthotopic PyMT-B6 mammary tumors and tumor-free controls, four samples consisting of two mice each were analyzed per group. (**e**) ISH for *Csf3* on end-stage PyMT-B6, KPC and tumor-free bone marrow tissue; n=3 per group. (**f**) Number of BM pre-DCs, CD24+ cDC1s and immature granulocytes and blood pre-DCs from mice bearing end stage PyMT-B6 GCSFKO clone 2 mammary tumors relative to tumor-free controls, n=6/group. End-stage is defined in the methods. Error bars represent mean +/- s.e.m.; *p<0.05, **p<0.01, ***p<0.001, n.s. not significant by unpaired two-sided Student's t test.

a.

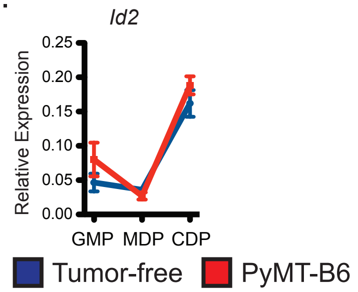

b.

IRF8 Regulated vs. Tumor Regulated	*p*-Values
All Genes	9.996E-12
Up-Regulated Genes	2.529E-08
Down-Regulated Genes	1.85E-08

c. d.

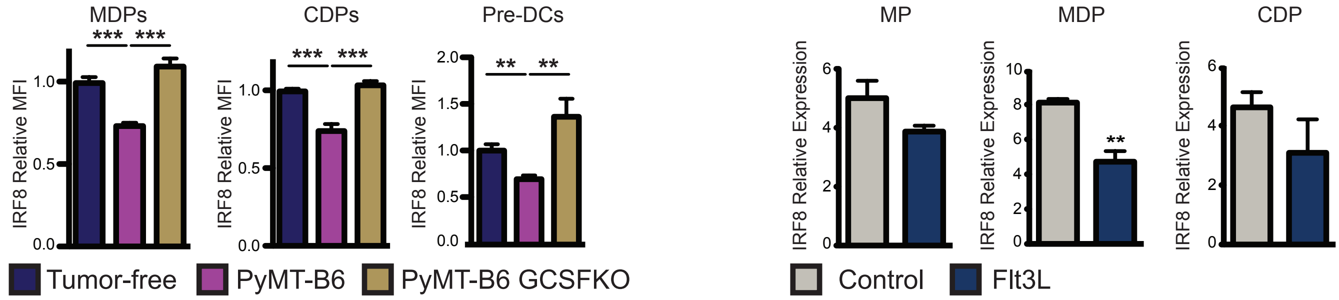

Supplementary Figure 6. (**a**) RT-qPCR analysis of BM GMPs, MDPs, and CDPs sorted from mice bearing end-stage orthotopic PyMT-B6 mammary tumors and tumor-free controls; four samples consisting of two mice each were analyzed per group. (**b**) Hypergeometric test of microarray from Fig. 3d comparing genes differentially expressed in IRF8$^{-/-}$ granulocyte progenitors and common myeloid progenitors generated in Waight et al. and Becker et al.[1,2] to genes differentially expressed in MDPs and CDPs from mice bearing end-stage orthotopic PyMT-B6 mammary tumors. End-stage is defined in the methods. (**c**) Relative IRF8 expression in BM MDPs, CDPs and pre-DCs from mice bearing end stage orthotopic PyMT-B6 or PyMT-B6 GCSFKO mammary tumors relative to tumor-free controls, n=6/group. (**d**) MPs, MDPs and CDPs treated with 100ng/ml Flt3L or media alone for 24 h. Analyzed by RT-qPCR for Irf8. Data are representative of two independent experiments consisting of three wells per condition. End stage is defined in the methods. Error bars represent mean +/- s.e.m.; *p<0.05, **p<0.01, ***p<0.001 by unpaired two-sided Student's t test.

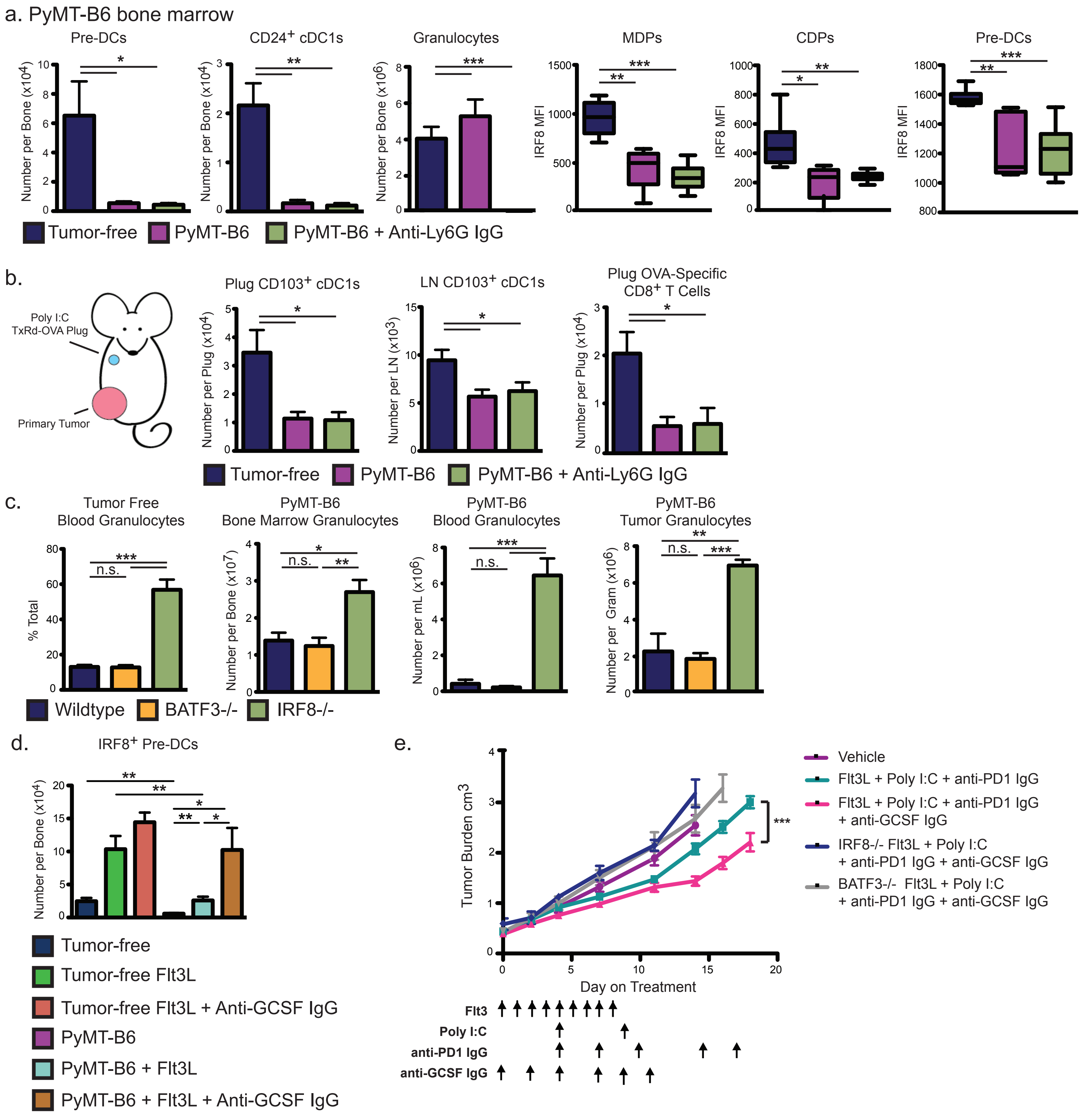

Supplementary Figure 7. (**a**) Tumor-free, orthotopic PyMT-B6 tumor-bearing end-stage mice and orthotopic PyMT-B6 tumor-bearing end-stage mice treated for 2 weeks with anti-Ly6G IgGs were analyzed. Numbers of BM pre-DCs, CD24$^+$ cDC1s, and granulocytes and IRF8 expression in BM MDPs, CDPs and pre-DCs; n=6/groups. (**b**) Mice with 1.0-cm diameter orthotopic PyMT-B6 tumors, mice with 1.0-cm diameter orthotopic PyMT-B6 tumors treated with anti-Ly6G IgGs, and tumor-free controls were implanted with matrigel plugs containing poly I:C TxRd-OVA peptide in the upper mammary fat pad. Number of CD103$^+$ cDC1s in the plug and draining LN and OVA-specific CD8+ T cells (CD3$^+$CD8$^+$Dextamer$^+$) in the plug 10 days after implant; n=5-6/group. (**c**) Percentage of granulocytes in the blood of tumor-free wild-type, BATF3-/-, and IRF8-/- mice. Number of granulocytes in the BM, blood, and tumor of wild-type, BATF3-/-, and IRF8-/- mice with end-stage PyMT-B6 orthotopic mammary tumors; n=6/group. (**d**) Number of IRF8$^+$ pre-DCs in tumor-free mice or orthotopic PyMT-B6 tumor-bearing end-stage mice treated for 2 weeks with 50 μg anti-GCSF IgGs 3x/week and/or 30 μg Flt3L daily for 9 days. (**e**) Tumor volume over time in established 1cm+ diameter orthotopic PyMT-mCh-OVA mammary tumors were treated with vehicle or Flt3L (30μg) + anti-PD1 IgGs (200 μg) + intratumoral Poly I:C (50 μg) +/- anti-GCSF IgGs (50ng) according to the displayed treatment schedule. End-stage for each model is defined in the methods. Error bars represent mean +/- s.e.m.; *p<0.05, **p<0.01, ***p<0.001, n.s. not significant by unpaired two-sided Student's t test or two-way ANOVA.

Supplementary Table 1: Breast Cancer Patient Demographic Data

			Breast Cancer Cohort 1		Breast Cancer Cohort 2	
**Age**						
Median			48		51	
Range			37-59		30-66	
**ER**	**PR**	**Her2**	**Count**	**%**	**Count**	**%**
-	-	-	3	30	11	52%
+	-	-	0	0	1	5%
-	+	-	1	10	1	5%
-	-	+	2	20	4	19%
+	+	-	3	30	3	14%
+	+	+	1	10	1	5%

Supplementary Table 2. MDP PyMT-B6 versus Tumor-Free

Gene	Fold-Change	p-value	Gene	Fold-Change	p-value	Gene	Fold-Change	p-value
Slfn14-ps	-14.199222	4.84E-05	H2afy	-1.757561	8.40E-05	Pabpc1l	3.164352	5.23E-04
9430091E24 Rik	-11.692082	4.99E-04	Rac3	-1.750934	2.32E-04	Srgap1	3.372656	3.59E-05
Cd209a	-11.247988	1.66E-06	Ifi203	-1.734656	1.49E-04	Mrgpra6	3.886934	2.47E-06
Srgap3	-8.818617	2.02E-04	Bcl2	-1.705624	6.63E-04	Lcn2	4.437278	1.76E-05
Eif2c4	-7.766489	2.31E-04	5830469G19 Rik	-1.654133	3.77E-04	5330421C15 Rik	4.760432	7.46E-04
1700084E18 Rik	-7.163278	2.15E-04	Mtr	-1.551082	3.01E-04	Abi3	4.815156	8.93E-05
Zfhx3	-6.163341	2.81E-04	St3gal2	-1.536023	5.69E-04	Lrg1	5.486857	2.51E-04
Pyroxd2	-5.797396	7.81E-05	Lst1	-1.521638	7.42E-04	Vldlr	6.371575	3.59E-04
Setdb2	-5.329231	5.67E-04	Map3k1	-1.44448	5.25E-04	Raet1c	7.024351	4.45E-04
Olfr1537	-4.702727	5.14E-04	Dph3	1.683221	6.54E-04	Hopx	7.591526	2.79E-04
Sema3c	-4.558992	3.57E-04	Ntpcr	1.7743	7.69E-05	Retnlg	7.682535	2.00E-04
Tom1l2	-4.454783	7.15E-04	Ptpn1	1.870916	4.03E-04	Cd300lf	7.738921	3.47E-04
AI607873	-4.184333	6.49E-04	Gm13315	1.987459	5.27E-04	1100001G20 Rik	8.626782	1.59E-06
Gm7265	-3.72731	3.85E-05	Emilin2	2.000381	3.20E-05	Chi3l3	9.473319	1.92E-05
Olfr516	-3.337054	6.92E-05	Rbm27	2.020952	3.02E-04	2810429I04 Rik	10.254729	1.82E-04
Fndc9	-3.305939	3.19E-04	Ptpn1	2.088149	4.85E-04	Cttnbp2nl	10.361098	1.24E-04
Ifi27l2a	-3.240912	3.61E-04	Cartpt	2.121081	2.51E-04	C030014L02	11.267027	1.27E-05
Pigm	-2.992684	4.80E-04	Ctsc	2.128556	1.81E-04	Olfm1	11.26855	1.23E-04
1700026L06 Rik	-2.724752	7.12E-04	Mapk1	2.160215	2.79E-04	Prg2	13.261688	5.10E-04
Tgtp2	-2.682215	4.28E-04	Pnp2	2.195014	1.89E-04	Spink2	15.657777	1.19E-05
Kcng1	-2.57158	7.03E-04	Lfng	2.249454	7.68E-05	Raet1c	3.164352	5.23E-04
4930469K13 Rik	-2.553343	5.33E-04	Ntpcr	2.343655	1.87E-04	Hopx	3.372656	3.59E-05
Dnahc8	-2.528804	6.95E-06	1110008P14 Rik	2.377219	1.44E-07	Retnlg	3.886934	2.47E-06
2900005J15 Rik	-2.522331	3.58E-04	Rab34	2.39526	3.26E-04	Cd300lf	4.437278	1.76E-05
Prkd2	-2.360954	5.62E-04	Ifitm1	2.491777	3.85E-05	1100001G20 Rik	4.760432	7.46E-04
Tnfsf12	-2.14273	7.54E-05	Dcdc2a	2.508631	5.14E-04	Chi3l3	4.815156	8.93E-05
Arhgap32	-2.103002	5.10E-04	Ifitm1	2.64112	3.72E-05	2810429I04 Rik	5.486857	2.51E-04
B930049P21 Rik	-2.023145	2.44E-05	Gda	2.763229	5.21E-04	Cttnbp2nl	6.371575	3.59E-04
Plin5	-2.013171	1.94E-04	Mrgpra2b	2.843982	4.88E-04	C030014L02	7.024351	4.45E-04
Pdzd4	-1.927993	1.32E-04	Ctsa	2.958713	2.73E-04	Olfm1	7.591526	2.79E-04
E330018D03 Rik	-1.925489	5.28E-04	Cyb561	3.007907	8.23E-05	Prg2	7.682535	2.00E-04
Padi2	-1.898487	7.95E-04	Prss16	3.018601	5.52E-04	Spink2	7.738921	3.47E-04
Smox	-1.883805	3.95E-04	Pim1	3.028021	1.67E-04			

Supplementary Table 3. CDP PyMT-B6 versus Tumor-Free (top 50 up-regulated and down-regulated genes)

Gene	Fold-Change	p-value	Gene	Fold-Change	p-value	Gene	Fold-Change	p-value
Cd209a	-32.729508	1.29E-04	Srgap3	-6.532035	8.55E-06	Trem3	4.8168	5.70E-06
Klrd1	-23.532619	4.26E-05	LOC101055889	-6.530269	6.66E-04	Ms4a6d	4.978876	3.77E-04
Gm6934	-23.027087	4.63E-05	Zkscan17	-6.526853	3.21E-04	Timp1	5.121362	1.57E-04
Trp53i11	-20.385374	2.12E-04	Enah	-6.519008	2.75E-04	Dmkn	5.29911	8.35E-06
Kdm5b	-15.639934	5.68E-06	Trp53i11	-6.435523	9.28E-06	Slc36a2	5.635418	2.83E-04
Cd96	-13.871963	1.56E-04	Mmel1	-6.022855	1.88E-05	Dmkn	5.707168	4.04E-06
2310042D19Rik	-12.788166	2.95E-06	Lrrc16a	-5.876094	9.28E-05	Ccm2l	5.779491	6.84E-04
Atp1b1	-12.443151	5.70E-05	Ngfr	-5.781675	5.40E-04	Lcn2	5.947259	2.43E-06
Nedd4	-12.340873	5.25E-09	Epb4.1l4b	-5.726684	7.26E-05	Gda	6.134507	1.40E-06
Gm15800	-12.188786	5.88E-04	Stap1	-5.606644	2.88E-04	Tgm1	6.261898	2.28E-06
Cd209e	-11.918143	1.15E-07	Rab37	-5.470566	3.33E-04	Fabp7	6.700668	7.62E-04
Upk1b	-11.861833	3.36E-06	Zeb1	-5.458445	1.99E-04	Hopx	7.01721	3.96E-04
Erg	-11.662276	2.36E-05	Lrrc16a	-5.247663	1.16E-04	Cd300lf	7.111838	5.02E-04
Snn	-11.626662	3.57E-04	Tnfrsf25	-5.083078	7.33E-05	Asns	7.210553	1.86E-06
Tspan2	-11.194533	6.23E-05	Clec1a	-5.078403	9.38E-05	Cd38	7.213915	2.23E-04
Kcnd1	-10.701696	2.34E-04	Zfp507	-4.959467	6.53E-04	Selp	7.260757	1.15E-04
Cd209a	-10.084709	2.82E-06	Cadm3	-4.901115	7.82E-04	Ccm2l	7.419001	2.59E-05
Srgap3	-9.856634	1.27E-04	Pla2g7	3.880863	6.85E-05	Clec5a	8.024478	1.64E-04
Spib	-9.443456	5.66E-04	Il18rap	3.907484	1.26E-06	Raet1c	8.333529	2.10E-04
Runx2	-8.458973	6.63E-06	Cd300lf	3.965554	1.27E-07	Ighv1-77	8.617922	2.80E-04
Zbtb46	-8.273169	7.10E-04	Agpat9	3.986306	5.84E-05	F11r	9.585708	5.52E-05
Siglech	-8.271463	2.90E-04	Dio2	4.007146	2.05E-04	Socs3	11.528477	2.69E-05
Tcf4	-8.160853	2.04E-04	Tctex1d1	4.062943	9.08E-07	BB031773	13.17378	2.62E-04
Jakmip1	-8.107488	5.06E-04	Ifitm1	4.182093	2.45E-07	Vcan	15.487367	3.60E-04
Soga1	-8.028317	2.99E-04	Ms4a3	4.185935	1.03E-06	Lrg1	15.686483	1.68E-06
Bend5	-8.019112	4.01E-04	Raet1c	4.191902	7.02E-04	a	16.804754	1.84E-05
Pid1	-7.696753	1.48E-05	Zfy1	4.264015	6.23E-04	Il22ra2	18.240837	2.23E-07
Mmel1	-7.620589	2.41E-05	Ifitm1	4.370657	3.44E-07	Vcan	21.760314	2.24E-08
Slfn14-ps	-7.277447	7.07E-04	Vcan	4.424341	1.62E-05	1100001G20Rik	26.74176	9.03E-09
Bach2	-7.08096	1.27E-04	Abi3	4.425031	1.52E-04	Il1r2	27.912414	7.08E-06
4933427I18Rik	-6.723465	1.99E-04	Irf2bpl	4.526874	6.57E-04	Clec4e	30.562592	1.50E-07
Cd46	-6.689739	1.89E-04	Mt1	4.574931	1.38E-07	Rgcc	41.203215	4.73E-05
E130215H24Rik	-6.532476	5.18E-07	Asb16	4.705131	2.28E-04	Crispld2	65.760643	5.57E-07

Supplementary Table 4. Enriched Biologic Process Terms

Enriched Biologic Process Term	*p*-Value
**Up-regulated Genes**	
Innate Immunity	1.8E-04
Secreted	2.7E-04
Innate Immune Response	1.3E-03
Myeloid Cell Differentiation	1.9E-03
Positive Regulation of Cytokine Secretion	3.8E-03
Defense Response to Bacterium	8.7E-03
Positive Regulation of Cell Death	9.9E-03
Cellular Response to Extracellular Stimulus	1.7E-02
Regulation of Cell Cycle	2.0E-02
Lipopolysaccharide-mediated Signaling Pathway	3.3E-02
Inflammatory Response	4.1E-02
**Down-regulated Genes**	
Transcriptional Regulation	4.2E-05
Transcription	8.1E-05
Covalent Chromatin Modification	7.0E-04
Homeostasis of Number of Cells Within a Tissue	8.1E-03
MHC Class II Protein Complex Binding	1.2E-02
Transcription Factor Binding	1.6E-02
Negative Regulation of Antigen Processing and Presentation of Peptide Antigen via MHC Class II	2.2E-02
Differentiation	2.8E-02
Histone Deacetylase Binding	3.8E-02

Supplementary Table 5. Human Flow Cytometry Antibodies

Antigen	Clone	Company	Color	Dilution
CD3	HIT3a	BD	PE	1:5
CD14	M5E2	BD	PE	1:5
CD19	HIB19	BD	PE	1:5
CD66b	G10F5	BD	PE	1:20
CD45	HI30	BD	BUV395	1:20
CD141	1A4	BD	BV510	1:20
CD1c	F10/21A3	BD	PerCP-Cy5.5	1:20
HLA-DR	G46-6	BD	PE-CF594	1:20
CD117	104D2	BD	BV605	1:20
CD116	hGMCSFR-M1	BD	BV421	1:100
CD135	A2F10.1	BD	APC	1:20
CD45RA	HI100	BD	BUV737	1:20
CD115	9-4D2-1E4	BD	BB515	1:20
Live/Dead Fixable Dead Cell Stain		Molecular Probes	Aqua	1:1000
Fixable Viability Stain		BD	FVS780	1:1000
CD45	HI30	eBioscience	Alexa Fluor 700	1:20
HLA-DR	L243	Biolegend	PE-Cy7	1:20
CD14	M5E2	BD	APC-Cy7	1:20
CCR2	K036C2	Biolegend	PerCP	1:20
CSF1R	9-4D2-1E4	Biolegend	PE	1:20
CD11b	ICRF44	Biolegend	Alexa 488	1:20
CD15	W6D3	Biolegend	Pacific Blue	1:20
CD16	3G8	Biolegend	APC	1:20
CD33	WM53	BD	BV510	1:20
CD1c	HI30	BD	PE	1:20
CD141	1A4	BD	PE	1:5
CD335	F10/21A3	BD	PE	1:5
CD10	G46-6	BD	PE	1:5
CD303	201A	Biolegend	PE	1:20
CD123	9F5	BD	BV395	1:20
CD34	581	Biolegend	BV510	1:20
IRF8	V3GYWCH	eBioscience	PerCP Cy5.5	1:200

Supplementary Table 6. Mouse Flow Cytometry Antibodies

Antigen	Clone	Company	Color	Dilution
CD45	30-F11	eBioscience	APC-eFluor780	1:400
CD45	30-F11	eBioscience	PE-Cy7	1:400
CD11b	M1/70	eBioscience	Alexa Fluor 700	1:400
CD11b	M1/70	eBioscience	Alexa Fluor 488	1:100
CD3e	145-2C11	eBioscience	PerCP-Cy5.5	1:200
CD3e	145-2C11	eBioscience	APC	1:200
CD11c	N418	eBioscience	APC-eFluor780	1:50
MHCII (I-A/I-E)	M5/114.15.3	eBioscience	eFluor450	1:400
Ly6G	1A8	Biolegend	PE	1:400
Ly6G	1A8	Biolegend	APC	1:400
Ly6G	1A8	Biolegend	FitC	1:400
CD8	53-6.7	eBioscience	FitC	1:400
CD8	53-6.7	BD	PE-CF594	1:400
Ly6C	HK1.4	eBioscience	PerCP-Cy5.5	1:400
GR1	RB6-8C5	Biolegend	Alexa Fluor 700	1:200
CD19	eBio1D3	eBioscience	APC	1:200
CD19	eBio1D3	eBioscience	Alexa Fluor 700	1:100
F4/80	BM8	eBioscience	PE-Cy5	1:400
CD16/32	93	eBioscience	Unconjugated	1:200
Ovalbumin MHCI Dextramer		Immudex	FitC	1:10
p-Stat3	D3A7	Cell Signaling	Alexa 488	
CD103	2E7	eBioscience	FitC	1:50
CD103	2E7	Biolegend	BV510	1:50
B220	RA3-6B2	Biolegend	APC	1:100
cKit (CD117)	2B8	Biolegend	PE-Cy7	1:200
Sca-1	D7	Biolegend	APC-Cy7	1:100
Flt3 (CD135)	A2F10.1	BD	PE-CF594	1:20
Sirpα (CD172α)	P84	Biolegend	FitC	1:100
Sirpα (CD172α)	P84	Biolegend	PerCP Cy5.5	1:100
IRF8	V3GYWCH	eBioscience	PerCP-eFluor710	1:50
CSF1R (CD115)	AFS98	eBioscience	PE	1:50
CSF1R (CD115)	AFS98	Biolegend	BV605	1:200
CD45.1	A20	eBioscience	APC	1:50
CD45.1	A20	Biolegend	FitC	1:100
CD45.2	104	Biolegend	FitC	1:200
CD45.2	104	Biolegend	APC	1:200
Streptavidin		Biolegend	APC	1:200
Lineage Cocktail		Miltenyi Biotec	Biotin	1:10
CD34	RAM34	eBioscience	eFluor450	1:10
CD16/32	93	eBioscience	PE	1:200
Ter-119	TER-119	eBioscience	APC	1:200
Siglec H	eBio440c	eBioscience	PE-Cy7	1:100

Supplementary Table 7. Markers Used for Flow Cytometry

Cell Type	Species	Tissue	Markers
**All Cells Pre-Gated on Singlets and Live Cells**			
GMP	Human	BM	Lin^{-}CD123^{-}cKit^{+}CD34^{+}GMCSFR^{-} Flt3^{+}CD45Ra^{+}MCSFR^{-}
MDP	Human	BM	Lin^{-}CD123^{-}cKit^{+}CD34^{+}GMCSFR^{-} Flt3^{+}CD45Ra^{+}MCSFR^{+}
CDP	Human	BM	Lin^{-}CD123^{+}cKit^{+}CD34^{+}GMCSFR^{+}MCSFR^{-} CD45RA^{+}
Pre-DC	Human	BM/Blood	CD45^{+}GMCSFR^{+}Flt3^{+}SSC^{lo}MCSFR^{-}CD45RA^{+}
CD141^{+} cDC1	Human	BM	CD45^{+}CD3^{-}CD14^{-}CD19^{-}CD66b^{-}Flt3^{+} HLA-DR^{+}CD141^{+}CD1c^{-}
CD1c^{+} cDC2	Human	BM	CD45^{+}CD3^{-}CD14^{-}CD19^{-}CD66b^{-}Flt3^{+} HLA-DR^{+}CD141^{-}CD1c^{+}
Immature Granulocytes	Human	BM/Blood	CD45^{+}CD11b^{+}CD33^{Hi}CD14^{-}CD15^{+}
Granulocyte	Human	Bone Marrow	CD45^{+}CD11b^{+}CSF1R^{-}CD15^{+}CD14^{-}
MP	Mouse	Bone Marrow	Lin^{-}cKit^{+}Sca1^{-}
GMP	Mouse	Bone Marrow	Lin^{-}cKit^{+}Sca1^{-}CD34^{+}CD16/32^{+}
MDP	Mouse	Bone Marrow	Lin^{-}CD11c^{-}MHCII^{-}Flt3^{+}MCSFR^{+}cKit^{Hi}
CDP	Mouse	Bone Marrow	Lin^{-}CD11c^{-}MHCII^{-}Flt3^{+}MCSFR^{+}cKit^{Mid}
Pre-DC	Mouse	Bone Marrow/Blood	Lin^{-}CD11c^{+}MHCII^{lo} Sirpα^{lo}Flt3^{+}
Pre-cDC1	Mouse	Bone Marrow	Lin^{-}CD11c^{+}Flt3^{+}cKit^{+}Zbtb46-GFP^{+}
CD24^{+} cDC1	Mouse	Bone Marrow	Lin^{-}Flt3^{+}MHCII^{+}CD11c^{+}CD24^{+}Sirpα^{-}
Sirpα^{+} cDC2	Mouse	Bone Marrow	Lin^{-}Flt3^{+}MHCII^{+}CD11c^{+}CD24^{-}Sirpα^{+}
CD103^{+} cDC1	Mouse	Tumor/Plug	CD45^{+}Ly6C^{-}MHCII^{+}F4/80^{-} CD24^{+}CD103^{+}CD11b^{-}
CD11b^{+} cDC2	Mouse	Tumor	CD45^{+}Ly6C^{-}MHCII^{+}F4/80^{-}CD24^{+}CD103^{-} CD11b^{+}
CD103^{+} cDC1	Mouse	Lymph Node	B220^{-}CD11c^{+}MHCII^{Hi}CD103^{+}CD11b^{-}
CD8α^{+} cDC1	Mouse	Spleen	B220^{-}CD11c^{+}MHCII^{+}CD8^{+}CD11b^{-}
Inflammatory Monocytes	Mouse	Spleen	CD45^{+}CD11b^{+}CSF1R^{+}Ly6G^{-}Ly6C^{+}
Resident Monocyte	Mouse	Spleen	CD45^{+}CD11b^{+}CSF1R^{+}Ly6G^{-}Ly6C^{-}
Immature Granulocytes	Mouse	Bone Marrow	CD45^{+}Lin^{-}CD11b^{+}MCSFR^{+}Ly6G^{+}
Granulocyte	Mouse	Bone Marrow	CD45^{+}Lin^{-}CD11b^{+}MCSFR^{-}Ly6G^{+}
Granulocyte	Mouse	Blood	CD45^{+}CD11b^{+}MCSFR^{lo}Ly6G^{+}
(OVA-Specific) CD8^{+} T Cell	Mouse	Tumor/Plug/Lymph Node	CD3^{+}CD8^{+}(Dextamer^{+})
Plasmacytoid DCs	Mouse	Bone Marrow/Blood	CD19^{-}CD11b^{-}B220^{+}SiglecH^{+}MHCII^{Mid}CD11c^{Mid}
cDC1	In Vitro		MHCII^{+}CD11c^{+}CD24^{+}Sirpα^{-}
Granulocytes	In Vitro		CD11b^{+}Ly6G^{+}Ly6C^{-}

Supplementary Table 8. RT-qPCR Primer Sequences from Applied Biosystems

**Target Gene**	**Assay ID**
Irf8	Mm00492567_m1
Cd209a	Mm00460067_m1
Zbtb46	Mm00511327_m1
Bcl11a	Mm00479358_m1
Spi1	Mm00488140_m1
SpiB	Mm03048233_m1
Cebpe	Mm02030363_s1
Gfi1	Mm00515853_m1
Cebpa	Mm00514283_s1
Id2	Mm00711781_m1
Batf3	Mm01318274_m1
H2-Ke6	Mm00469140_g1
H2-Dma	Mm00439226_m1
H2-Q8	Mm02394722_gH
Tapbp	Mm00493417_m1
Tap2	Mm01277033_m1
Pml	Mm00476969_m1
Csf3r	Mm00432735_m1
Tbp	Mm00446971_m1
Gapdh	Mm99999915_g1

SUPPLEMENTARY REFERENCES

1. Waight, J.D., et al. Myeloid-derived suppressor cell development is regulated by a STAT/IRF-8 axis. J Clin Invest **123**, 4464-4478 (2013).
2. Becker, A.M., et al. IRF-8 extinguishes neutrophil production and promotes dendritic cell lineage commitment in both myeloid and lymphoid mouse progenitors. Blood **119**, 2003-2012 (2012).
